# Laser ablation-based one-step generation and bio-functionalization of gold nanoparticles conjugated with aptamers

**DOI:** 10.1186/1477-3155-8-21

**Published:** 2010-08-23

**Authors:** Johanna G Walter, Svea Petersen, Frank Stahl, Thomas Scheper, Stephan Barcikowski

**Affiliations:** 1 Institut für Technische Chemie, Leibniz Universität Hannover, Callinstrasse 3, 30167 Hannover, Germany; 2 Laser Zentrum Hannover, Hollerithallee 8, 30419 Hannover, Germany

## Abstract

**Background:**

Bio-conjugated nanoparticles are important analytical tools with emerging biological and medical applications. In this context, *in situ *conjugation of nanoparticles with biomolecules via laser ablation in an aqueous media is a highly promising one-step method for the production of functional nanoparticles resulting in highly efficient conjugation. Increased yields are required, particularly considering the conjugation of cost-intensive biomolecules like RNA aptamers.

**Results:**

Using a DNA aptamer directed against streptavidin, *in situ *conjugation results in nanoparticles with diameters of approximately 9 nm exhibiting a high aptamer surface density (98 aptamers per nanoparticle) and a maximal conjugation efficiency of 40.3%. We have demonstrated the functionality of the aptamer-conjugated nanoparticles using three independent analytical methods, including an agglomeration-based colorimetric assay, and solid-phase assays proving high aptamer activity. To demonstrate the general applicability of the *in situ *conjugation of gold nanoparticles with aptamers, we have transferred the method to an RNA aptamer directed against prostate-specific membrane antigen (PSMA). Successful detection of PSMA in human prostate cancer tissue was achieved utilizing tissue microarrays.

**Conclusions:**

In comparison to the conventional generation of bio-conjugated gold nanoparticles using chemical synthesis and subsequent bio-functionalization, the laser-ablation-based *in situ *conjugation is a rapid, one-step production method. Due to high conjugation efficiency and productivity, *in situ *conjugation can be easily used for high throughput generation of gold nanoparticles conjugated with valuable biomolecules like aptamers.

## Background

Gold nanoparticles (AuNPs) feature unique optical properties, including high surface plasmon resonance (SPR), enhanced absorbance and scattering with high quantum efficiency. In addition to their resistance against photobleaching, AuNPs perfectly fulfill requirements for use as colorimetric sensors and markers. For sensing or labeling of DNA targets, AuNPs can fairly easily be functionalized with DNA via thiol linkers, resulting in a highly ordered, self-assembled monolayer (SAM)[1,2]. Numerous colorimetric applications of DNA-conjugated AuNPs have already been developed[3].

More recently, several applications of aptamer-conjugated AuNPs have been reported[4,5]. Aptamers are short, single-stranded DNA or RNA molecules that exhibit high specificity and affinity towards their corresponding target. Thus, aptamers can be thought of as nucleic acid analogues to antibodies that can be selected *in vitro *via SELEX (systematic evolution of ligands by exponential enrichment) against virtually any molecule, including proteins as well as small molecules like metal ions[6-8]. Aptamer-conjugated AuNPs have already been successfully used for the detection of proteins in a dry-reagent strip biosensor,[9] for detection of thrombin on surfaces,[4] for colorimetric detection of platelet-derived growth factor,[5] for detection of adenosine and potassium ions in an agglomeration-based approach,[10] for detection of thrombin in a dot blot assay[11] and for targeting and therapy of cancerous cells[12,13].

All applications of aptamer-conjugated AuNPs published so far have been based on chemical synthesis of AuNPs in the presence of reducing and stabilizing agents, and subsequent (*ex situ*) ligand exchange with aptamers. This ligand exchange might require heating and buffering in order to achieve satisfactory yields and surface coverage. The latter might be limited by interference from remaining reducing agents with the aptamer during the replacement process. Additionally, remaining precursors and/or reducing agents might result in a possible restriction of AuNPs use in biomedical applications[14,15].

Recently, laser ablation of gold in a liquid environment has been used for the production of AuNPs[16,17], using surfactants for growth quenching, resulting in narrow nanoparticle size distributions[18]. The advantages of laser-generated AuNPs include high purity in combination with unique surface characteristics. The Au surface of laser-generated AuNPs is partially oxidized, resulting in electrostatic stabilization of the colloid without the need for chemical additives. These partially positively-charged AuNPs, acting as electron acceptors, can interact directly with electron donors like amino or thiol groups in the ablation medium[19,20]. During laser ablation, the DNA acts as a capping agent, allowing precise size control of the resulting AuNPs, as it has been previously reported for the addition of cyclodextrines, biopolymers, etc[21]. Recently, a direct comparison of conventional *ex situ *conjugation of laser-generated AuNPs and laser-ablation-based *in situ *conjugation of AuNPs with DNA has revealed a four times higher conjugation efficiency when using the laser-ablation-based procedure. In comparison to AuNPs produced by chemical synthesis and subsequent *ex situ *conjugation, AuNPs generated using laser-ablation-based *in situ *conjugation exhibit up to five times higher surface coverage[22]. Hence, bio-conjugation during laser ablation presents a rapid and efficient preparation method, especially for the conjugation of valuable biomolecules like aptamers or vectors. The high surface coverage of DNA-modified AuNPs produced by *in situ *conjugation may be especially advantageous for applications including cellular uptake of AuNPs. In this context, Giljohann *et al*. have found that the extent of cellular uptake of DNA-modified AuNPs can be increased by enhancing the DNA loading[23]. Moreover, high DNA densities can also facilitate cooperative binding, resulting in increased association constants with a given target, e.g. in intracellular gene regulation[24]. Another important parameter that can be modulated via surface coverage is the immune response induced by DNA-modified AuNPs. Higher DNA densities efficiently limit the immune response as measured by Interferon-β expression in mouse macrophages[25].

In spite of these benefits, the use of laser-ablation-based *in situ *conjugation for the generation of aptamer-conjugated AuNPs has not yet been reported.

We show the functionalization of nanoparticles with aptamers during femtosecond-pulsed, laser-induced gold nanoparticle formation in an aqueous media using a DNA aptamer directed against streptavidin as a model system. In order to demonstrate the applicability of aptamer-conjugated AuNPs generated via laser ablation in complex biomedical applications, we have used an RNA aptamer directed against prostate-specific membrane antigen (PSMA) for the detection of PSMA in human prostate cancer tissue utilizing tissue microarrays.

## Results and Discussion

### Choice of aptamer orientation and spacer design

In order to ensure aptamer activity, several factors concerning the ability of the aptamer to fold into the correct three-dimensional structure have been considered. We have previously reported the application of an aptamer directed against streptavidin (referred to as miniStrep) in a protein microarray format[26,27]. Using this approach, we have found that the miniStrep aptamer requires an additional spacer placed between the aptamer and the substrate to show activity that is slightly higher when immobilized via its 3' terminus. Thus, we decided to use 3' orientation. An additional oligothymidine (T10) spacer was placed between the disulfide group and the aptamer sequence. Tymidine was chosen, since this nucleotide has the lowest affinity towards the gold surface[28]. Thus, nonspecific binding of the spacer bases to gold is minimized, which should increase the surface loading and improve elevation of the aptamer away from the nanoparticle surface. Taking these considerations into account, the miniStrep aptamer construct used in this work was the following: TCT GTG AGA CGA CGC ACC GGT CGC AGG TTT TGT CTC ACA G -T_10_-(CH_2_)_3_-S-S-(CH_2_)_6_OH.

We decided to immobilize the anti-PSMA aptamer via the 3'terminus. According to Lupold *et al*., the aptamer can be subjected to 3' truncation of up to 15 nucleotides without losing its affinity to PSMA[29]. Since the 3' terminal bases are not necessary for target recognition, we decided to omit the use of an additional oligonucleotide spacer. Instead, hexaethylenglycol was chosen as a spacer, because it does not exhibit intermolecular repulsion, which is one cause of low DNA loading on AuNPs[30]. Furthermore, it only occupies a small surface area, which allows high packing densities,[30] and is known to minimize nonspecific protein binding[31]. Therefore, the aptamer construct used was the following: GGG AGG ACG AUG CGG AUC AGC CAU GUU UAC GUC ACU CCU UGU CAA UCC UCA UCG GCA GAC GAC UCG CCC GA-(CH_2_CH_2_O)_6_-(CH_2_)_6_-S-S-(CH_2_)_6_OH.

**Figure 1:**
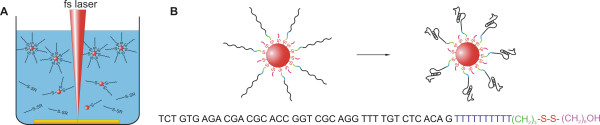
**Generation of aptamer-conjugated AuNPs via *in situ *conjugation**. (A) Schematic illustration of *in situ *conjugation of AuNPs with aptamers during laser ablation in an aqueous aptamer solution. (B) Spacer design and resulting mixed monolayer conjugated nanoparticles. Mixed monolayer formation and careful spacer design contribute to correct aptamer folding.

The aptamers were directly used in the laser ablation process without prior dithiothreitol (DTT) treatment (Figure 1A). According to Dougan *et al*., this does not affect surface coverage[32]. Moreover, the mercaptohexanol (MCH) of the mixed disulfide (aptamer-S-S-(CH_2_)_6_OH) may serve as a co-adsorbent, eliminating unspecific binding to the gold surface, by occupying free binding sites[33]. Due to the formation of a mixed monolayer consisting of aptamers and short organic residues, the available space for optimal aptamer folding is enhanced (Figure 1B).

### *In situ *conjugation

**Figure 2:**
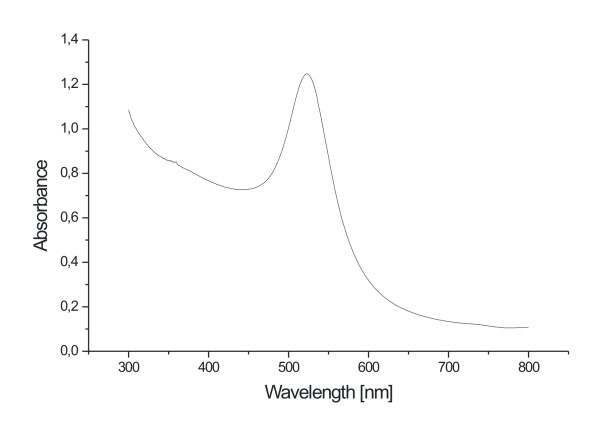
**UV/VIS spectrum of aptamer-conjugated AuNPs**. The spectrum was obtained with an as prepared AuNP solution after *in situ *conjugation with 5 μM anti-streptavidin aptamer.

Due to rapid, one-step processing, laser-ablation-based *in situ *conjugation enables fast screening of different conjugation conditions. Utilizing this high throughput potential, we have determined optimal conjugation conditions by using different concentrations of miniStrep aptamer in a Tris(hydroxymethyl)-aminomethan (Tris) buffer during laser ablation. Per investigated concentration, the laser ablation process took less than two minutes. A UV/VIS spectrum of AuNPs produced via laser ablation in the presence of 5 μM aptamer can be found in Figure 2.

**Figure 3:**
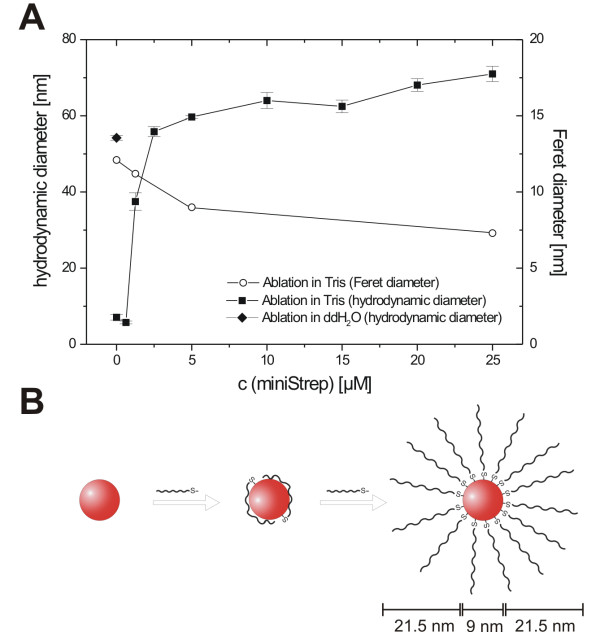
**Characterization of aptamer-conjugated AuNPs**. (A) Hydrodynamic diameter and Feret diameter of the aptamer AuNP conjugates as a function of the aptamer concentration used during laser ablation. (B) Suggested mechanism of the observed increase of the size of the conjugates. At low surface coverage, the negatively charged aptamer lays flat on the positively charged AuNP surface. With increasing surface coverage, the aptamers straightened up on the surface, resulting in an increased hydrodynamic diameter. Scale bars illustrate the proportions of linearized aptamer and AuNP.

DLS measurements demonstrate that the hydrodynamic diameter (d_h_) of the AuNPs increases with increasing aptamer concentrations (Figure 3A). While d_h _after ablation in Tris buffer (without aptamer) is 7 nm, d_h _increases with increasing aptamer concentrations up to 5 μM, and finally reaches a plateau of approximately 60 - 70 nm (Figure 3A). We assume that this d_h _increase is a result of cumulative aptamer loading on the gold surface. At low surface coverage, the aptamer lays flat on the surface, due to non-specific binding via the lone nitrogen electron pairs of the nucleotides. As the surface coverage increases, the aptamers are forced to adopt a more perpendicular conformation, due to electrostatic repulsion of the aptamers' negatively charged phosphate backbones, resulting in a d_h _increase (Figure 3B). We estimated the length of the aptamer (including T_10 _spacer) to be 21.5 nm, using a base to base distance for ssDNA of 0.43 nm[34]. For an aptamer-conjugated AuNP of 9 nm core size, this results in a diameter of approximately 52 nm, which is close to the observed plateau of d_h_, and supports our assumption (Figure 3B).

On first sight, the AuNPs hydrodynamic diameter increase seems to be contradictory to our previous finding of a growth quenching effect induced by increasing DNA concentrations[19]. But in contrast to our previous work, here the ablation was performed in Tris buffer. Tris interacts with the surface of the embryonic AuNPs, resulting in prevention of further post-ablation nanoparticle agglomeration. Consequently, the AuNPs produced in Tris buffer are already stabilized by the buffer molecule, resulting in reduced diameters (d_h_= 7.1 ± 0.8 nm in Tris buffer versus 54.2 ± 0.6 nm in ddH_2_O (Figure 3A)) and a diminished influence of the oligonucleotide concentration on the nanoparticle size. This assumption is supported by TEM analysis data. The Feret diameter (d_Feret_) of AuNPs produced in Tris buffer slightly decreases from 12.1 to 7.3 nm with increasing aptamer concentration (Figure 3A). In comparison to the Tris molecule, the thiolated aptamer exhibits a higher affinity towards the gold surface, resulting in better stabilization of embryonic particles, and thus smaller AuNPs, as detected via TEM analysis. Although there may be some portion of Tris-aptamer ligand exchange after nanoparticle generation, the size quenching effect observed by TEM analysis confirms successful *in situ *bio-conjugation during laser ablation.

In addition to the AuNP size, we have determined aptamer loading (Table 1). For AuNPs produced by laser ablation in a 5 μM aptamer solution (*in situ*), we found a loading of 98 aptamers per nanoparticle, corresponding to 65 pmol/cm^2^. This aptamer loading is higher than the results achieved by post production (*ex situ*) modification of chemically synthesized AuNPs with short oligonucleotides (Demers *et al*.: 34 pmol/cm^2^),[35] and the aptamer loading to chemically synthesized AuNPs reported by Huang *et al*. (13 pmol/cm^2^)[5]. The high aptamer loading achieved by *in situ *conjugation confirms high availability of laser-generated AuNPs for bio-conjugation. The conjugation efficiency was calculated as the portion of provided aptamer bound to the nanoparticle surface (Table 1). At a 1.25 μM aptamer concentration, 40.3% of the available aptamer binds to the AuNPs, which demonstrates the suitability of the method for efficient conjugation of valuable biomolecules. Since we have observed no denaturation of the aptamer during laser ablation (as discussed in the next section), the remaining aptamer can be reused.

**Table 1:** Characterization of aptamer-conjugated gold nanoparticles

**c**_**(miniStrep)**_ [μM]	**d**_**h **_**(DLS)**^***a***^ [nm]	**d**_**Feret**_^***b***^ [nm]	Aptamer/AuNP	**Aptamer/A**_**AuNP**_^***c***^ **[pmol/cm**^**2**^**]**	**E**_**con**_^***d***^ [%]
**0**	7.1 ± 0.8	12.1 ± 9.8	-	-	-

**1.25**	37.5 ± 2.3	11.2 ± 4.2	80 ± 2	33.93 ± 1.04	40.3 ± 0.3

**5**	61.1 ± 1.9	9.0 ± 5.0	98 ± 4	64.58 ± 1.82	19.8 ± 0.7

**25**	71.0 ± 2.0	7.3 ± 2.5	74 ± 11	73.90 ± 7.22	6.1 ± 0.9

Summary of data obtained for AuNPs conjugated with miniStrep aptamer in Tris buffer (^*a *^Hydrodynamic diameter, ^*b *^Feret diameter, ^*c *^Aptamers per AuNP surface, ^*d *^Conjugation efficiency).

After the ablation process, conjugates were slowly transferred into the aptamer selection buffer by adding NaCl and MgCl_2_. During this salting process, we observed precipitation of AuNPs produced at aptamer concentrations lower than 5 μM. The higher stability of AuNPs conjugated at aptamer concentrations of 5 μM (or higher) coincides with the plateau in the hydrodynamic diameter (Figure 3B), and indicates better stabilization due to higher surface coverage.

**Figure 4:**
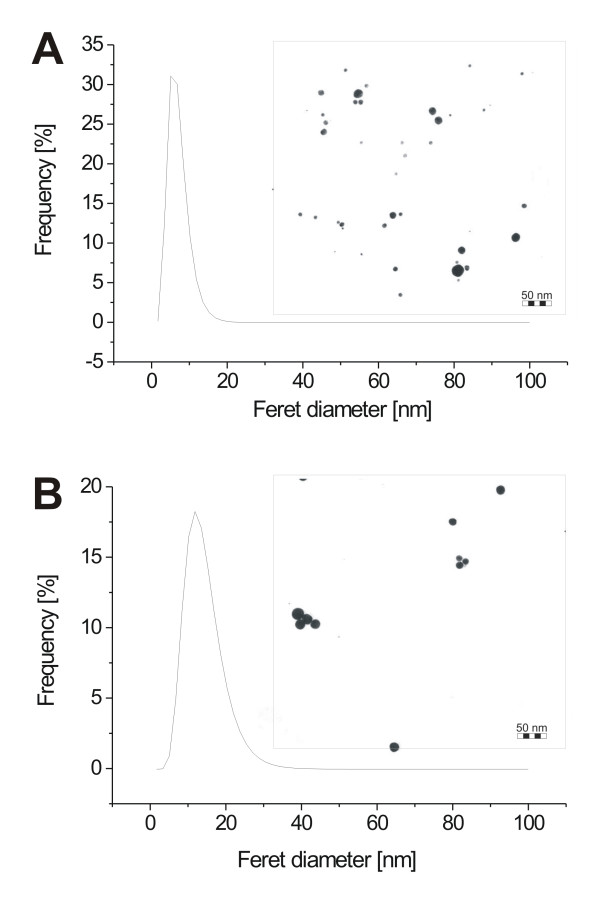
**TEM analysis of aptamer-conjugated AuNPs**. TEM micrographs and AuNP size distributions (lognormal fit) of AuNPs produced by laser ablation in 5 μM miniStrep solution before (A), and after (B) removal of free aptamer using centrifugation.

All further experiments were performed with AuNPs produced in a 5 μM aptamer solution, which was a compromise between maximal aptamer density and minimal aptamer consumption (Table 1). Under these conditions, we could produce 75 μg (150 μg/ml) miniStrep-conjugated AuNPs in less than two minutes. The free aptamer was removed by centrifugation. In order to maintain conjugate activity, centrifugation was performed under rather mild conditions (16600 × g). This procedure results in a slightly increased average Feret diameter (14.6 nm) due to loss of small nanoparticles (Figure 4).

It should be noted that the high immobilization efficiency, and thus high aptamer consumption, results in decreasing aptamer concentrations during the laser ablation process. As we suppose that the aptamer conjugation takes place in the millisecond to second regime after the collapse of the cavitation bubble, the aptamer loading of NPs will also decrease over this period of time. If more homogeneous aptamer loadings are required, this can be achieved by applying higher aptamer concentrations and/or by shortening ablation time.

### Functionality of miniStrep-conjugated AuNPs

**Figure 5:**
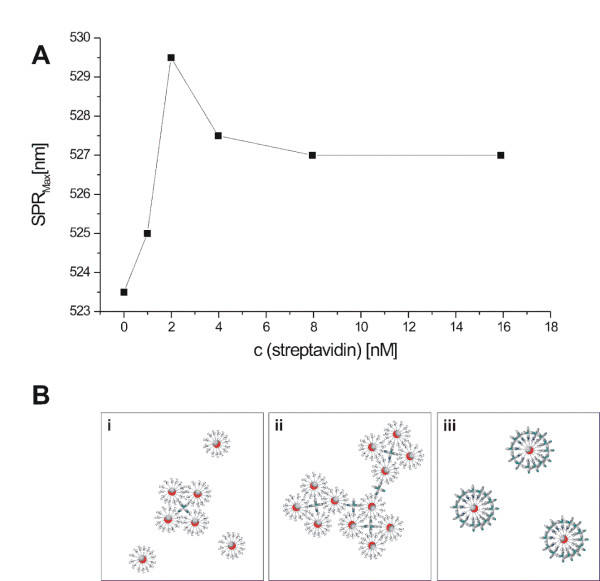
**Verification of the activity of aptamer-conjugated AuNPs**. (A) The shift of the SPR maximum clearly indicates the formation of agglomerates in the presence of streptavidin. (B) Schematic illustration of the formation of agglomerates as a function of streptavidin concentration: At low streptavidin concentrations, relatively small agglomerates occur (i). At medium streptavidin concentrations, the tetrameric protein induces the formation of large agglomerates (ii). An excess of streptavidin inhibits the formation of agglomerates by saturation of the aptamers bound to the nanoparticle surface (iii).

**Figure 6:**
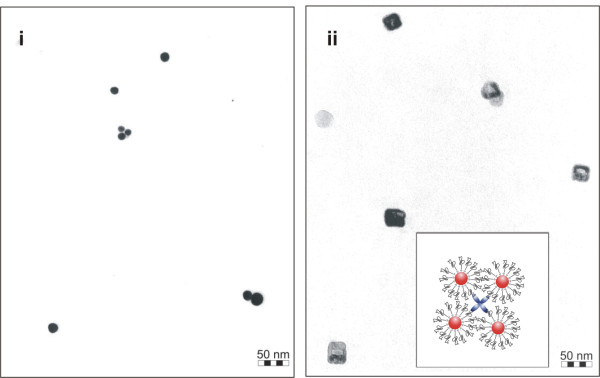
**TEM analysis of AuNPs conjugated with aptamers against streptavidin**. TEM micrographs of AuNPs without streptavidin (i), and after incubation with streptavidin (ii). The insert displays a scheme of the proposed composition of the agglomerates. Please note that the agglomerates are displayed in a simplified manner in the scheme, de facto streptavidin is not planar but tetrahedral.

Functionality of the immobilized miniStrep aptamer was confirmed by using three independent methods. First, a classical, agglomeration-based method was applied. A fixed amount of AuNPs (0.69 nM) conjugated with miniStrep aptamer was incubated with different amounts of streptavidin (0 - 15.9 nM), and UV/VIS was detected. Since streptavidin is a tetrameric protein, agglomeration can be observed as a red shift of SPR_Max _(Figure 5A). The shift in SPR_Max _increases with increasing concentrations of streptavidin, and reaches a maximum at a streptavidin concentration of 2 nM. In addition to the SPR_Max _shift, we observed the formation of a red film on the wall of the reaction vessel at streptavidin concentrations from 1 nM to 4 nM. Simultaneously, we observed a loss of AuNPs in the solution. Based on absorbance at 380 nm, the loss of particles was calculated to be 86.5%. We assume that the red film is composed of large agglomerates, while small agglomerates stay in the solution and can be detected via the shift of SPR_Max _and TEM analysis. TEM micrographs of the agglomerates indicated a defined composition and tetrahedral structure of these agglomerates (Figure 6). Based on TEM analysis, we determined an agglomerate size of 35 nm (edge-to-edge length). In order to verify the proposed tetrahedral structure, we calculated the size of the agglomerates based on the observed shift of SPR_Max_, utilizing the "plasmon ruler equation":[36]

$$\frac{\Delta \lambda }{\lambda_{0}}\approx 0,18\times \exp\left(\frac{-\left(\frac{s}{D}\right)}{0,23}\right)$$

This approximation describes the dependency between the observed shift of SPR_Max _(Δλ) on the interparticle gap (s) and nanoparticle size (D). Using our experimental results (Δλ = 6 nm; λ0 = 523.5 nm; D = d_Feret _= 14.6 nm), we calculated an interparticle distance of 9.3 nm, and an edge-to-edge length of the proposed tetrahedron of 38.5 nm. Taking into account that equation (1) is an empirical approximation established for a pair of interacting nanoparticles rather than for a tetramer, and considering that the geometry of streptavidin is not perfectly tetrahedral, the deviation of 10% between the agglomerate sizes measured by TEM analysis and calculated from the shift of SPR_Max _seems to be acceptable. Good agreement between the agglomeration sizes obtained using two independent methods supports the proposed tetrahedral structure of the agglomerates.

At streptavidin concentrations above 2 nM, the SPR_Max _shift decreases, due to saturation of aptamers immobilized on the AuNPs surface with streptavidin (Figure 5B). This saturation effect is in accordance with the observations of Huang *et al*., who used an aptamer against a dimeric protein (platelet-derived growth factor)[5].

In order to gain quantitative insight into streptavidin binding and thus aptamer activity, the AuNPs were incubated with an excess of Cy3 labeled streptavidin. The agglomerates of aptamer-coated nanoparticles and attached streptavidin were removed using ultracentrifugation, and the amount of bound streptavidin was determined by measuring the remaining streptavidin concentration in the supernatant.

Since aptamer loading was determined for the whole AuNP population generated by laser ablation (d_Feret _= 9.0 nm), and the binding of Cy3 labeled streptavidin was performed with the AuNP subpopulation resulting from removal of the free aptamer by centrifugation (d_Feret _= 14.6 nm), it was not possible to compare aptamer loading directly to the amount of bound streptavidin. In order to estimate the aptamer activity, it was assumed that aptamer density (pmol/cm^2^) is not significantly affected by the nanoparticle diameter. Based on this assumption, the aptamer loading was calculated to be 64.58 ± 1.82 pmol/cm^2^, and the amount of streptavidin bound to the AuNP surface was 67.23 ± 0.76 pmol/cm^2^, resulting in approximately 100% aptamer activity. This indicates that the aptamer is not degraded during the laser ablation process, and optimal aptamer folding was achieved by careful design of the spacer.

**Figure 7:**
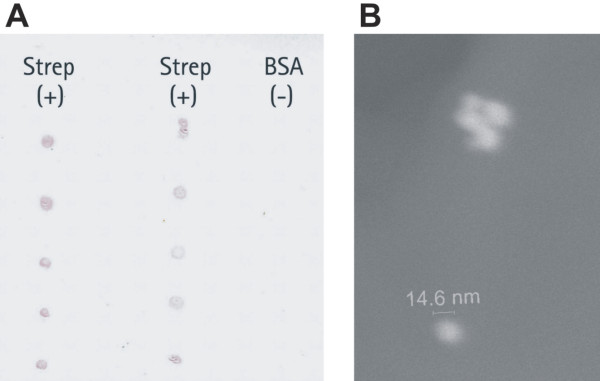
**Dot blot**. Dot blot detection of streptavidin, using aptamer-conjugated gold nanoparticles (A). ESEM image of nanoparticles bound to streptavidin immobilized on the nitrocellulose membrane (B).

To examine the applicability of the aptamer-conjugated AuNPs in solid phase assays, we performed a simple dot blot assay (Figure 7A). Within this assay, BSA was used as a negative control. BSA was chosen because it exhibits a mildly acidic isoelectric point (pI) similar to the pI of streptavidin (pI 5-6) resulting in a comparable negative net charge of the two proteins under the given conditions (pH 7.4). In order to exclude the possibility of electrostatic interactions between the negatively charged proteins and the positively charged AuNPs, the experiment was repeated with unconjugated nanoparticles. The nanoparticle aptamer conjugates bind only to the immobilized streptavidin, and no binding can be observed to BSA. Using unconjugated nanoparticles, no binding of AuNPs to the immobilized proteins occurred (data not shown). This clearly demonstrates the specific binding of AuNPs to streptavidin via the aptamer conjugated to the nanoparticle surface.

The AuNPs bound to streptavidin during the dot blot assay were further analyzed via ESEM. The ESEM micrograph affirms the Feret diameter of the nanoparticles determined by TEM (Figure 7B).

### Functionality of anti-PSMA-conjugated AuNPs

Encouraged by the positive performance of the dot blot assay, our next aim was to prove the applicability of aptamer-conjugated AuNPs in more complex and demanding solid-phase assays. Therefore, we used AuNPs conjugated with an aptamer directed against PSMA for detection of PSMA in prostate cancer (adenocarcinoma) tissue sections.

**Figure 8:**
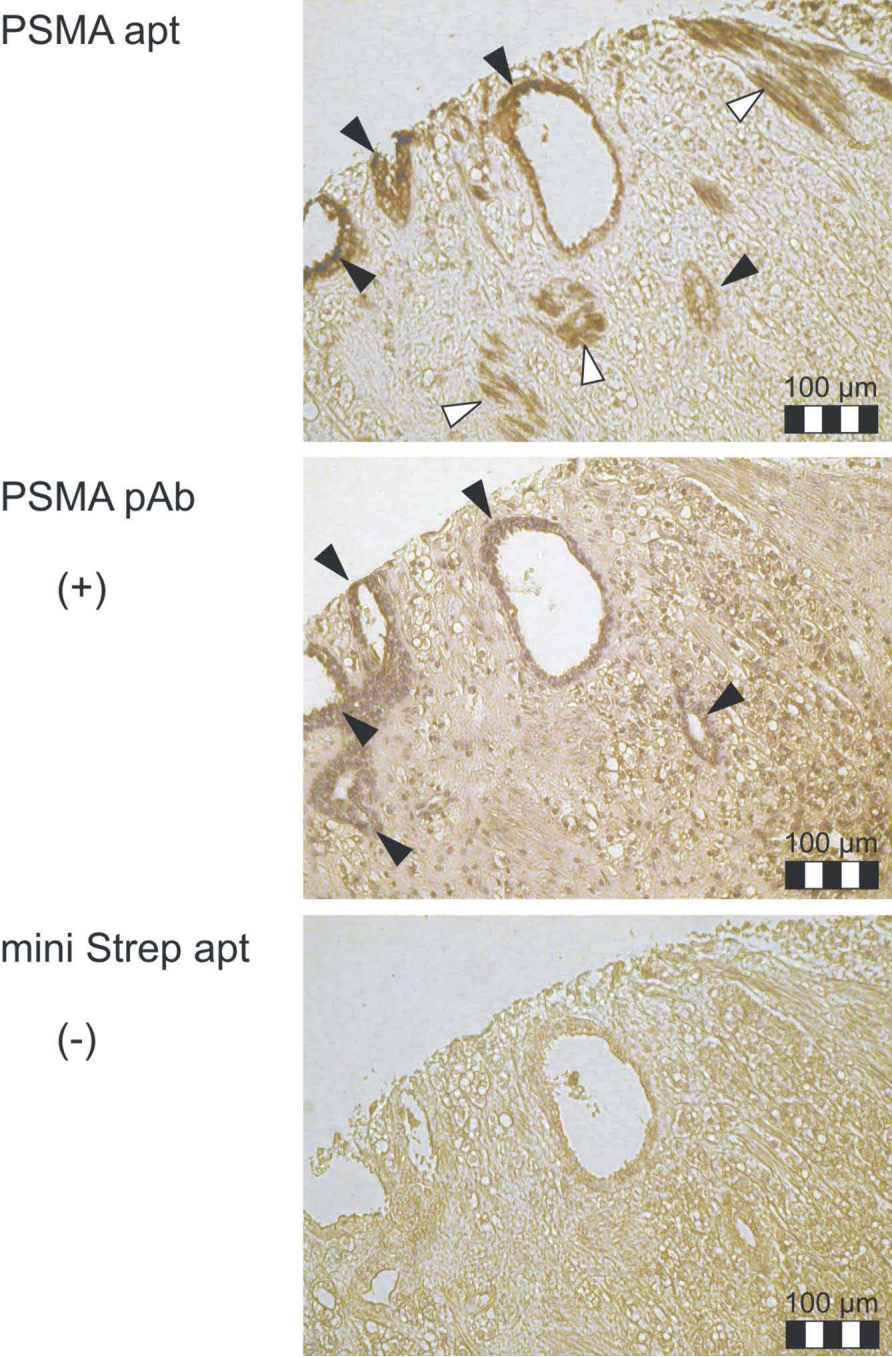
**Detection of PSMA in human prostate cancer tissue**. Detection of PSMA positive structures in prostate cancer tissue sections by immunohistochemical staining using anti-PSMA aptamer (PSMA apt)-conjugated AuNPs. As a negative control, AuNPs conjugated with miniStrep aptamer (miniStrep apt) were used. A polyclonal antibody directed against PSMA (PSMA pAb) was used as a positive control. Positive control was additionally stained with Haematoxylin and Eosin. Black arrows indicate specific staining, while white arrows flag unspecific binding.

AuNPs conjugated with anti-PSMA aptamer show a staining pattern similar to anti-PSMA antibody. In both cases, a positive staining of acinar epithelial cells was observed (Figure 8). In tissue sections treated with anti-PSMA aptamer-conjugated AuNPs, an additional staining of muscle cells was observed that was not detected in the positive control. To ensure that the binding to PSMA is based on the affinity of the anti-PSMA aptamer rather than on electrostatic interaction between the target protein and the highly negatively charged aptamers, AuNPs conjugated with anti-streptavidin aptamers were used. Since the negative control does not show positive binding to epithelial cells or false positive binding to muscle cells, we assume the binding of anti-PSMA conjugates to muscle cells to be induced by the specific three-dimensional structure of the anti-PSMA aptamer. A positive staining of smooth muscle cells in prostate cancer has also been reported for one monoclonal PSMA antibody (7E11),[37] and some authors assume that there may be a "PSMA-like" target in smooth muscle cells[38,39]. Following this consideration, the binding of AuNPs conjugated with anti-PSMA aptamer to muscle cells may be the result of cross-reactivity of the aptamer with this unknown "PSMA-like" target. In summary, our results demonstrate that the anti-PSMA aptamer AuNP conjugates can detect PSMA in acinar epithelial cells of human prostate cancer. This exemplifies the broad applicability of aptamer-conjugated AuNPs, even in highly complex biological matrices and bio-imaging applications.

## Conclusions

We have demonstrated the suitability of laser-ablation-based *in situ *bio-conjugation for the production of functional, aptamer-conjugated gold nanoparticles. Exploiting the potential of this rapid, one-step method for high throughput screening, we have optimized the conjugation regarding aptamer loading and conjugation efficiency. To address the general applicability of the method, we have utilized two different aptamers composed of DNA and RNA. The high degree of aptamer activity determined on AuNP surface verifies that there is no heat-induced denaturation of the aptamer during laser ablation. We have proven the functionality of conjugates using three different methods (agglomeration-based assay, dot blot assay, tissue microarray), indicating the broad applicability of aptamer-conjugated gold nanoparticles for bio-analytical applications, even in highly demanding assays. Moreover, *in situ *conjugation avoids possible contamination by toxic educts, residual reducing agents or preservatives. Thus, this method could also be especially advantageous for use in medical applications.

Since *in situ *conjugation is a fast and simple one-step approach to generate pure conjugated AuNPs with high conjugation efficiency and productivity, it can be easily used for the high throughput production of large amounts of different conjugated nanoparticles. The higher conjugation efficiencies are beneficial for high-priced biomolecules, and the comparably high surface coverage is desirable for cellular uptake, which depends on the DNA density on the AuNPs surface. Moreover, such high surface densities may assist cooperative binding and may decrease the immune response against AuNPs.

## Methods

### Materials

All chemicals were purchased from Sigma-Aldrich (Steinheim, Germany) or Fluka Chemie AG (Taufkirchen, Germany), and used as received. The aptamer against streptavidin (TCT GTG AGA CGA CGC ACC GGT CGC AGG TTT TGT CTC ACA G -T_10_-(CH_2_)_3_-S-S-(CH_2_)_6_OH, referred to as miniStrep) [26] and anti-PSMA aptamer (GGG AGG ACG AUG CGG AUC AGC CAU GUU UAC GUC ACU CCU UGU CAA UCC UCA UCG GCA GAC GAC UCG CCC GA-(CH_2_CH_2_O)_6_-(CH_2_)_6_-S-S-(CH_2_)_6_OH) [29] were purchased from Biospring GmbH (Frankfurt, Germany). The gold foil was 0.1 mm thick and had >99.99% purity, and was obtained from Goodfellow GmbH (Bad Nauheim, Germany).

### Generation of aptamer-conjugated AuNPs

Laser ablation was performed in the same buffer system the aptamer was originally selected in. For the miniStrep aptamer, 50 mM Tris(hydroxymethyl)-aminomethan (Tris) pH 8.0 was used, and the anti-PSMA aptamer was conjugated in 20 mM N'-2-Hydroxyethylpiperazine-N'-2 ethanesulphonic acid (HEPES) pH 7.4. Laser generation of AuNPs was performed utilizing a Spitfire Pro femtosecond laser system (Spectra-Physics) providing 120 fs laser pulses at a wavelength of 800 nm. 5×5 mm gold foils were placed in the wells of a 24 well plate filled with 500 μl of aptamer solution in the respective buffer. Ablation was performed while moving the plate at a constant speed of 60 mm×min-1 in a spiral (outer radius: 3 mm, inner radius: 1.5 mm), using an axis system. Recently, we have optimized the laser parameters for laser-ablation-based generation of DNA-conjugated AuNPs[19]. Here, laser fluence was optimized in regard to maximal productivity, while avoiding degradation of the oligonucleotide. In the present study, the optimized parameters were chosen, the pulse energy was fixed at 100 μJ, and the repetition rate was 5 kHz. In order to avoid heat-induced degradation of the aptamer, the focus position was adjusted to be 2 mm beneath the focus position determined in air[19].

### Post-generation processing of the aptamer-conjugated AuNPs

After laser ablation, the conjugates were allowed to age overnight at 4°C before NaCl was added in increments of 25 mM by addition of 2 M NaCl in Tris-Cl or HEPES respectively. After each NaCl addition, the colloidal solution was mixed and incubated for 1 h at room temperature. The addition of MgCl_2 _and CaCl_2 _was performed after another overnight incubation at 4°C, by addition of 1 M MgCl_2 _and 1 M CaCl_2_. Final buffer compositions were the following: miniStrep: 150 mM NaCl, 10 mM MgCl_2_, 50 mM Tris-Cl pH 8.0; anti-PSMA: 150 mM NaCl, 1 mM MgCl_2_, 1 mM CaCl_2_, 0.05% Tween 20, 20 mM HEPES pH 7.4.

To remove the free aptamer, the ablation medium was centrifuged for 15 min at 15000 rpm. The supernatant was transferred into a new centrifugal tube and centrifuged for another 30 min. The supernatant was discarded, and the pellets were pooled and resuspended in the respective buffer. This process was repeated 4 times.

### Characterization methods

UV/VIS spectra of the AuNP solutions were recorded using a Shimadzu 1650 spectrophotometer. In order to determine the AuNP concentration, the absorption at 380 nm (mainly corresponding to the interband transition of gold) was measured. Intensities were converted to AuNP mass concentrations by interpolation from a linear standard calibration curve (R^2 ^= 0.99). Standard curves were prepared with known concentrations of AuNP produced by weighing a gold target three times before and after ablation.

Transmission electron micrographs (TEM) were commissioned at Stiftung Tierärztliche Hochschule, Institut für Pathologie (Prof. Dr. W. Baumgärtner, Kerstin Rohn), and were obtained by utilizing a TEM Philip CM30 with a 0.23 nm resolution. One drop of the colloidal solution was placed on a carbon-coated, formvar-covered copper grid, and then dried at room temperature. Given diameters were averaged for at least 200 AuNPs. Dynamic light scattering (DLS) measurements were performed, using a Zetasizer ZS (Malvern). Three consecutive measurements were carried out and average values are presented.

The amount of aptamer bound per nanoparticle was determined by measuring the concentration of the unbound aptamer. Aptamer-conjugated AuNPs were removed by ultracentrifugation (Beckman Coulter Optima Max, 30000 × g), and the adsorption of the supernatant was measured at 260 nm against a serial dilution of aptamer in Tris buffer. Mean values of three measurements are presented.

### Determination of miniStrep aptamer functionality

The agglomeration-based streptavidin assay was performed by incubating a fixed amount of miniStrep-conjugated AuNPs (0.69 nM) with varying concentrations of streptavidin (0 - 15.9 nM) for 16 h at room temperature. UV/VIS was measured to monitor the shift of SPR_Max_.

Furthermore, the aptamer activity was determined in a "golden blot"[40] format similar to the method published by Wang *et al*[11]. In brief, streptavidin (0.5 μl, 1 mg/ml in PBS) was spotted in 10 replicates onto a nitrocellulose membrane (Sartorius, Goettingen, Germany). After 1 h incubation at room temperature, blocking of the membrane was performed with 1% BSA in miniStrep selection buffer. The membrane was washed in the same buffer and incubated with a solution of AuNPs (20 μg/ml) for 2 h. Finally, the membrane was washed with miniStrep selection buffer. As a negative control, BSA (0.5 μl, 1 mg/ml in PBS) was spotted on the membrane. Furthermore, the experiment was repeated with "bare" AuNP produced in Tris buffer in the absence of aptamer. In this experiment, the miniStrep selection buffer was replaced by 50 mM Tris-Cl pH 8.0, in order to maintain colloidal stability of the non-stabilized nanoparticles. Environmental scanning electron microscopy (ESEM) of the membrane after incubation with AuNPs was performed with a Quanta 400 F (FEI, Eindhoven, Netherlands) in low vacuum conditions. A piece of membrane was placed on an aluminum holder and visualized without previous sputtering.

In order to determine the activity of the miniStrep aptamer bound to the AuNP surface, the conjugate (28.5 μg/ml, 0.15 nM) was incubated with Cy3-labeled streptavidin (166.7 μg/ml, 2.8 μM) for 16 h at room temperature, in the dark. The conjugates and bound streptavidin were removed by ultracentrifugation. The amount of streptavidin bound to the nanoparticles was determined by measuring the streptavidin concentration remaining in the supernatant, utilizing a Fluoroskan ascent fluorescence plate reader (Ex: 544 nm, Em: 590 nm). Mean values of 4 measurements are presented.

### Determination of anti-PSMA aptamer functionality

The activity of anti-PSMA aptamers conjugated to AuNPs was investigated, using a tissue microarray consisting of paraffin-embedded prostate cancer tissues (US Biomax, Rockville, MD, USA). After baking the slides at 60°C for 30 min, paraffin was removed using two washing steps in xylene (10 min each). The tissue arrays were rehydrated by consecutive washes in 100%, 95% and 70% ethanol, followed by a washing step in ddH_2_O (5 min each). Antigen retrieval was performed by placing the slides in 0.01 M sodium citrate pH 6.0 for 15 min at 95°C. Consequently slides were washed with anti-PSMA aptamer selection buffer, and blocked in 5% goat serum (Millipore) in the same buffer. The anti-PSMA selection buffer was used for all consequent assay steps. Incubation with the aptamer-modified AuNPs (20 μg/ml) was performed for 2 h at 20°C and 300 rpm in an Eppendorf shaker equipped with a slide adaptor, after placing a secure seal incubation chamber (Grace Biolabs, Bend, OR, USA) filled with 800 μl of the respective AuNP solution on the slide. Slides were washed two times for 5 min with 1% goat serum, and fixed for 15 min with 2.5% glutaraldehyde solution. Silver enhancement was performed using a silver enhancer kit (Sigma), according to the instructions provided by the manufacturer.

AuNPs conjugated with miniStrep Aptamer in HEPES buffer were chosen as a negative control. All washing and incubation steps were performed as described above. As a positive control, a rabbit anti-PSMA antibody directed against the C-terminal domain of human PSMA (Millipore) was used[41]. Here, all washing and incubation steps were performed using PBS. After incubation with 2.5 μg/ml rabbit anti-PSMA for 2 h, the slides were washed two times for 5 min with 1% goat serum, and consequently incubated with a 1:20 dilution of 12 nm colloidal gold conjugated with goat anti-rabbit IgG (Jackson Immuno Research; OD at 520 nm of stock solution: 2) for 1.5 h. High background of developed tissue arrays was removed as described by Springall *et al*[42].

## Competing interests

The authors declare that they have no competing interests.

## Authors' contributions

JGW and SP carried out the *in situ *conjugations and partial drafting of the manuscript. JGW carried out the determination of aptamer functionality. JGW and FS carried out the tissue microarray experiments. SB carried out the principal study design, manuscript drafting and supervision of nanoparticle generation. TS participated in the conception design and supervised aptamer-related work. All authors read and approved the final manuscript.
